# Unraveling reproductive and maternal health challenges of women living with HIV/AIDS in Vietnam: a qualitative study

**DOI:** 10.1186/s12978-024-01768-3

**Published:** 2024-03-11

**Authors:** Lynn T. Nguyen, Le Minh Giang, Diep B. Nguyen, Trang T. Nguyen, Chunqing Lin

**Affiliations:** 1 David Geffen School of Medicine, University of California, Los Angeles, 855 Tiverton Dr, Los Angeles, CA USA; 2 Center for Training and Research On Substance Use and HIV, Hanoi Medical University, Room 211B, Building E3, No.1, Ton That Tung Street, Hanoi, Vietnam; 3 760 Westwood Plaza, 17-369E, Los Angeles, CA 90024 USA

**Keywords:** HIV/AIDS, Women, Stigma, Maternal care, Reproductive health, Vietnam

## Abstract

**Background:**

Human Immunodeficiency Virus (HIV) remains a significant public health concern worldwide. Women living with HIV/AIDS (WLHA) have the additional and unique need to seek sexual and reproductive health services. WLHA’s maternal health journeys can be shaped by the cultural norms and resources that exist in their society. This study sought to understand if and how WLHA’s family planning, pregnancy, and motherhood experiences could be influenced by the patriarchal culture, gender roles, and HIV stigma in Vietnam, specifically.

**Methods:**

Between December 2021 and March 2022, 30 WLHA with diverse socioeconomic backgrounds and childbirth experiences were interviewed in Hanoi, Vietnam. These semi-structured interviews covered topics including HIV stigma, gender norms, pregnancy experiences, and child-rearing challenges. Interviews were audio recorded, transcribed, and analysed using ATLAS.ti.

**Results:**

Qualitative analyses of participant quotes revealed how limited information on one’s health prospects and reproductive options posed a significant challenge to family planning. Societal and familial expectations as well as economic circumstances also influenced reproductive decision-making. WLHA often encountered substandard healthcare during pregnancy, labor, and delivery. Stigma and lack of provider attentiveness resulted in cases where women were denied pain relief and other medical services. Communication breakdowns resulted in failure to administer antiretroviral therapy for newborns. Motherhood for WLHA was shadowed by concerns for not only their own health, but also the wellbeing of their children, as HIV stigma affected their children at school and in society as well. Many WLHA highlighted the constructive or destructive role that family members could play in their childbirth decision-making and care-giving experiences.

**Conclusions:**

Overall, this study underscores the complex ways that cultural expectations, family support, and stigma in healthcare impact WLHA. Efforts to educate and engage families and healthcare providers are warranted to better understand and address the needs of WLHA, ultimately improving their reproductive and maternal health.

## Plain language summary

HIV-related stigma and discrimination have consequential impacts on health and quality of life for women living with HIV (WLHA). WLHA in Vietnam must navigate the additional challenges of a traditionally patriarchal and hierarchical society. Women typically face less educational and occupational opportunities and are often expected to defer to expectations of family and virtue. Stigma among family members, friends, employers, and healthcare providers poses a significant challenge to WLHA autonomy, especially as it relates to their reproductive health decision-making and maternal health experiences. This study aims to better understand the experiences of WLHA throughout family planning, pregnancy, and motherhood. The findings will hopefully shed light on strategies to empower WLHA and to combat HIV- and gender-based stigma not only in Vietnam, but also globally.

## Background

Human Immunodeficiency Virus (HIV) remains a significant public health concern worldwide, particularly for women. The latest estimates from the World Health Organization approximate that 38 million people are living with HIV/AIDS, with women accounting for 48% [1]. Researchers have recognized biological factors related to HIV acquisition, viral pathogenesis, and response to treatment which contribute to sex-specific differences in HIV-related morbidity and mortality [2, 3]. Socioeconomic factors such as gender-based financial disparities and educational inequities result in disproportionate access to healthcare and worse outcomes for women living with HIV/AIDS (WLHA) [4, 5]. Importantly, WLHA have the unique need to seek sexual and reproductive health services, and their maternal health journeys can be shaped by stigma, breach of confidentiality, and denial of care [6, 7].

In Vietnam, women constitute 33% of the 240,000 people living with HIV/AIDS [8]. Socio-cultural beliefs and gender norms in Vietnamese society shape stigma that arises from a belief that HIV infection is associated with immoral behaviors, such as sex work and intravenous drug use, which are considered “social evils” [9]. Confucian principles also hold great influence, prescribing a woman’s duty as a wife and her obligations to family [10]. These societal beliefs profoundly impact the decision-making and experiences of WLHA throughout their reproductive journey. The concept of filial piety establishes respect and authority for senior family members. Family can influence a couple’s decision to have children, with parents and in-laws often instilling pressure to produce a male heir [11]. This cultural dynamic can particularly affect WLHA in their family planning choices. When it comes to pregnancy, existing literature indicates delays in HIV testing and challenges in accessing optimal prevention of mother-to-child HIV transmission (PMTCT) services, often stemming from a lack of information and reluctance to discuss HIV-related issues [12, 13]. Furthermore, WLHA often face societal perceptions of being unfit for motherhood due to their illness and the risk of transmitting HIV to their children [14].

Despite significant impacts on the outcomes for WLHA, there is insufficient research on the intersection of HIV stigma, cultural beliefs, and gender roles that together shape multifaceted challenges WLHA face across the various stages of their reproductive journey. Most existing literature on WLHA in Vietnam centers around their pregnancy desire and perinatal services [10–15], revealing a gap in current research concerning the challenges WLHA experience as a mother. In light of changing cultural contexts and policies, the present study uses a qualitative method to explore continued challenges faced by WLHA throughout family planning, pregnancy, and motherhood as influenced by individual, familial, and social-cultural factors. The insights gained are intended to inform multilevel strategies that not only address the specific vulnerabilities of WLHA but also enhance their access to and engagement with overall reproductive health services.

## Methods

### Participant recruitment

Between December 2021 and March 2022, we conducted 30 in-depth qualitative interviews with WLHA in Hanoi, Vietnam. Participants were recruited from HIV outpatient clinics and community-based organizations serving women. Recruited WLHA were asked to invite female peers from the community to participate. Participation criteria were (1) age 18 or above, (2) assigned female sex at birth, and (3) HIV-seropositive status. To ensure a comprehensive sample, we selected WLHA with varying ages, marital status, educational attainment, employment, transmission route, antiretroviral therapy status, and number of children. Before the interview, participants were informed of the study purpose, procedures, potential risks and benefits, and that participation was voluntary. Oral informed consent was obtained. Approval to conduct this research was obtained from University of California, Los Angeles, and Hanoi Medical University.

### Participant characteristics

Of the 30 WLHA interviewed, 15 (50%) were 36–45 years old, 24 (80%) achieved some middle or high school education, and 21 (70%) had unstable employment. The majority of WLHA (66.7%) reported infection via a sexual route and 13.3% via needle sharing. At the time of interview, 13 (43%) WLHA were married or cohabited with a partner and 11 (36.7%) had a husband/partner also living with HIV. The WLHA participants were at different reproductive stages: three (10%) of the participants did not have children at the time of the study, 18 (60%) had one child, and nine (30%) had two children (Table 1).

**Table 1:** Participant characteristics (N = 30)

Characteristics	n	%
Age		
18–35	7	23.3
36–45	15	50.0
46 and above	8	26.7
Education		
Elementary school or below	5	16.7
Middle school	9	30.0
High school	15	50.0
College	1	3.3
Employment		
Stable job	7	23.3
Unstable job(s)	21	70.0
Unemployed	2	6.7
Transmission route		
Sexual	20	66.7
Needle sharing	4	13.3
Unclear	6	20.0
Marital status		
Married	13	43.3
Divorced or separated	5	16.7
Widowed	8	26.7
Single	4	13.3
Partners’ HIV status		
Positive	11	36.7
Negative	13	43.3
Unknown	3	10.0
N/A	3	10.0
Number of children		
0	3	10.0
1	18	60.0
2	9	30.0
Number of children after tested HIV +		
0	16	53.3
1	13	43.3
2	1	0.03
Having child(ren) tested HIV +		
Yes	2	6.7
No	28	93.3

### Data collection

The semi-structured interviews lasted 60–90 min in private settings. Considering participant preference and the COVID-19 situation, interviews were either in-person or virtual on Zoom. All interviewers had previous experience with qualitative research and received additional training regarding this study’s objectives and questions. The interview topics commenced with general discussions, covering areas such as cultural and personal beliefs related to HIV, as well as the social and familial roles of women in the context of HIV/AIDS. These conversations then progressed to more specific inquiries about the experiences and challenges faced by WLHA at various reproductive stages, from family planning, pregnancy, prenatal and postpartum care, to child-rearing. Personal identifiers were not collected. All participants were paid 300,000 VND (approximately 13 USD) for their participation.

### Data analysis

The interviews were audio recorded with participants’ consent. Interviews were transcribed verbatim in Vietnamese and translated into English. Data analysis was performed concurrently with participant recruitment and interviewing to inform data saturation. The data segments were organized into coding categories using ATLAS.ti (v3.15.0). A set of codes was developed based on the interview guides before examining the data. The developed codes were applied and modified based on themes that emerged from the transcripts throughout the coding procedure [16]. The coding was performed by a team of three PhD or Master level public health researchers. To ensure reliability, the coders read the transcripts, discussed analysis, and established inter-rater reliability by using rates of agreement. Themes relating to experiences, challenges, and support systems encountered during family planning, pregnancy, prenatal and postnatal care, and child caring, were identified and extracted.

## Results

### Family planning

Many factors played into a WLHA’s decision to have children, with the presence or absence of family and social support having the strongest influence. Due to deeply ingrained social expectations for women to continue their family bloodline, some WLHA feared being marginalized by their families, especially the in-laws, if they did not have children.

Without a child, they [husband’s family] still think that I’m like an outsider…She [mother-in-law] doesn’t really see me as her daughter. Firstly, I am sick. Secondly, I can’t give birth to children for their family. (Woman aged 50, middle school education, unstable employment, one adult child)

Many WLHA deferred to pressure from their husbands to bear children, even if their own health condition made it unfavorable, for the sake of preserving family unity.

I suffered a lot and was on the verge of death after giving birth to my first child. Moreover, I was addicted to drugs, so I didn’t know how to take care of myself. However, my husband insisted on keeping our child as he was the only son in the family, and he had no child. (Woman aged 41, high school education, unstable employment, two teenage children)

On the other hand, the absence of spousal or in-law support was a frequent deterrent for WLHA to have children. WLHA expressed concerns that, should their health declined, they lacked a stable family support system to care for their children.

A bad health condition or illness would prevent me from raising them. For this reason, I decided not to have kids. Now, I am even more certain of it. I am unable to have a stable family or husband and children. Having these things makes a woman feel secure. My current situation is… “Single mom” is what it’s called. (Woman aged 40, middle school education, unstable employment, one adult child)

Additionally, WLHA faced a significant challenge due to inadequate access to information and resources necessary for informed decision-making in family planning, leaving them in a state of despair and confusion about their available options and the prospects of pregnancy.

When I found out about my condition, I didn’t have anybody to share things with. I didn’t know any group or organization…and I didn’t know where to ask for help. I would have gotten an abortion because I fell into despair. I thought having this condition meant my life had already come to an end. (Woman aged 37, middle school education, unstable employment, two children: one teenager and one younger).

Finally, 77% of WLHA in this study (Table 1) were either unemployed or had unstable employment. Many did not have wealthy families to whom they could turn to for financial assistance. Without financial security, many WLHA worried that they could not adequately provide for their children.

Our economic situation means that we can’t provide the best conditions for our child. That’s the foremost reason for not having more, not because of the disease. So of course, it did affect my plan. I will not have another child. (Woman aged 35, high school education, unstable employment, one teenage child)

### Pregnancy and perinatal care

Stigma and discrimination posed significant barriers to WLHA’s autonomy in pregnancy and access to quality perinatal services. Several WLHA opted for abortion due to discouragement by medical providers. In an extreme case, one WLHA accepted forced sterilization due to fear of infecting future children.

I started taking the medicine that helped prevent HIV transmission to my baby. When I had my delivery…the doctors made me undergo sterilization. I accepted…I was afraid that my daughter would be infected with HIV, so I accepted the operation. (Woman aged 43, some elementary school education, unstable employment, two children: one adult and one teenager)

Being denied admission or referred to other healthcare settings was commonly experienced by WLHA when her HIV status was revealed. Providers there claimed lack of expertise in delivering babies born to mothers with HIV.

They [hospital staff] said there was nobody specializing in baby delivery in such circumstances [HIV positive mothers]. They advised me to go to the National Hospital instead. (Woman aged 37, middle school education, unstable employment, two children: one teenager and one younger)

During labor and delivery, several WLHA recalled being denied routine services such as pain relief due to discrimination from healthcare providers.

When I entered the delivery room, the doctors there discriminated against me. The doctors didn’t want to take care of me. There were many times when I had labor pain and I asked for an operation multiple times, but the doctors just ignored me. (Woman aged 36, high school education, stable job, one teenage child)

One WLHA explained how she was forced to undergo a caesarean section before her due date, without appropriate informed consent.

I was expected to be in labor in early December. That was my due date. However, after I had been diagnosed [with HIV], the doctor said, ‘I don’t care whether you will be in labor in early December. You will have a cesarean section next Monday. (Woman aged 44, high school education, unstable employment, one teenage child)

Another WLHA shared her experience of being left unattended. She delivered her baby without professional assistance due to healthcare staffs’ avoidance and fear of contracting HIV. Her husband had to step in and assist with the delivery.

They [the hospital staff] didn’t dare to touch me. It was in 2006 when people didn’t know much about my illness…At that time, while I was in pain, they told me to keep waiting, waiting, waiting... I gave birth right on the spot. My husband delivered my baby…no one would help me (Woman aged 41, high school education, unstable employment, two teenage children)

### Postpartum care and support

Stigma in healthcare settings continues to be a barrier for WLHA to receive optimal postpartum care. WLHA faced denial of postpartum medical services including bathing and wound care. Some family members had to step in and take responsibility for these tasks.

Most people knew that I got HIV so they wouldn’t bathe the baby. We just bathed our baby ourselves. Also, in that hospital, there were no wound care services…my family asked, they just said no. (Woman aged 36, high school education, unstable employment, two young children)

Additionally, a few WLHA underwent more costly medical procedures without proper explanation. For instance, one WLHA recognized the discrepancies in her medical bills compared to her peers. She shared how it intensified her stress as she, among many WLHA, was already struggling financially.

When it came to my operation, the woman who did my hospital papers said that I had to pay more to support the doctors, the operation team. But I saw that my friends… for this same thing...they paid 1 million dong, some 2 million, and some 3 million…but for me the deal was 7 million dong. 7 million is not a big amount of money, but for my husband and I, it was everything. There were more payments with paper signing, in total it was 11 million dong or so. (Woman aged 35, high school education, unstable employment, one young child)

A prominent factor affecting WLHA’s perinatal experiences was the lack of knowledge and information sources, which could be stemmed from failure of medical providers to provide WLHA with proper information and guidance. In some cases, there was a breakdown in communication and WLHA were blamed for not taking the necessary medical precautions for PMTCT.

It was after a month when I came to get her examinations done that the nurse asked me whether my daughter was taking medications. I told her that I didn’t know…The staff over at the hospital said that as soon as we went home after the birth, they suddenly remembered us. They said that I did not pick up the phone when they called. But no one was calling me. Those nurses said it was my fault! (Woman aged 29, college education, unstable employment, one young child)

There were WLHA in this study stressed that these knowledge and care gaps resulted in their babies getting infected; a consequence that would affect their lives forever.

They helped me deliver, and that’s all. There were no medications. There was nothing here back then. My baby got infected. After that time, I didn’t dare give birth. To date, I still have not given birth again. (Woman aged 44, high school education, unstable employment, one adult child)

Due to unclear guidance on what was safe to do as a new parent, many WLHA in this study lamented missing out on opportunities to physically bond with their newborns and felt that they were not caring for their child in a way that was natural and expected.

I purposely didn’t let my breast milk flow so that my son had to get milk from other sources, so that…there was no contact between us...I only knew not to breastfeed my baby, I didn’t really know much else. I just thought about ‘not being near my baby.’ (Woman aged 36, some high school education, unstable employment, one teenage child)

Experiences or fears of discrimination and isolation from their families have adversely affected the postpartum experiences of WLHA. In some cases, families avoided visiting them in the hospital or separated them from their babies. Additionally, concerns arose that their inability to breastfeed might inadvertently reveal their health status, leading to gossip and further stigmatization within the family.

### Childcaring and parenting

During the parenting stage, similar to their experiences in pregnancy and perinatal periods, the lack of knowledge among WLHA exacerbated fears about their health deteriorating, leading to concerns about the future care and well-being of their children.

I don’t know how long I will live. I’m also worried that there are many things I haven’t done for my children, I’m just afraid that my children are still small and if I get sick then I don’t know how they will live. (Woman aged 38, middle school education, stable employment, one teenage child)

The theme of family and social support, or lack thereof, remained important factors contributing to either successes or challenges of motherhood and childcare. Multigenerational households are a common cultural practice in Vietnam and a few WLHA expressed gratitude for having parents or in-laws who were willing to help with childcare when they needed. Many WLHA were also fortunate enough to receive instrumental emotional and financial support from their families.

At that time, I also had my biological mother, my biological mother took care of my son from A to Z…When I was sick like that, I had my mom take care of everything for my son. (Woman aged 36, some high school education, unstable employment, one teenage child)

Some WLHA deliberately disclosed their HIV status to certain family members, not only as an act of trust, but also as a pragmatic step to ensure that there would be someone informed and ready to step in to care for their children if their health took a turn for the worse.

I told my sister first. At that time, I thought I might not be able to live, so she could know my situation and my children…If I die, she will help take care of them for me. (Woman aged 45, some elementary school education, stable employment, two children: one adult and one teenager)

As a cultural expectation, WLHA faced the burden of raising their children alone without much involvement of their husbands/partners to share the childrearing burden. Several WLHA also shared the resentment they felt towards their partners for infecting them and explained how their strained relationships impacted how they raised their children.

It has been 17 years since my husband died. I raise my son on my own. I have been taking on different responsibilities for the past 19 years. My son is 19 years old, and I have been in charge of everything myself, completely alone…I raise my kid alone, teach him things alone. In general, I take care of everything, from A to Z, alone. (Woman aged 38, middle school education, stable employment, having one adult child)

Encouragingly, some WLHA used the fact that there was no other family around as motivation for living well and living long so that their children could have someone to care for them.

Sometimes I think about my [deceased] husband and I think about my child. If I die now, she will have a hard life, having no father and no mother…It’s not worth it to die because of HIV. I try to maintain positive thoughts and to live well so that my daughter won’t be ashamed of me. (Woman aged 55, elementary school education, unstable employment, one teenage child)

WLHA who use drugs faced additional challenges of raising young children while battling addiction without the support of family or friends. They did not have the socioeconomic resources to provide safe and nurturing home environments for their children.

There was only one tiny room (at home), if they (the children) didn’t stay with us, where would they go? When we were in withdrawal, we didn’t think about anything, we let the two small children sit and play next to us while we were on drugs, because no one was watching them for me. Once while I was using drugs, my children even played with a syringe (Woman aged 41, some high school education, unstable employment, two teenage children)

Finally, an important factor influencing WLHA’s experiences with motherhood was navigating the societal stigma and lack of social support that not only affected themselves, but also their children. Some WLHA described how their children were neglected or isolated by neighbors. Schools also required discriminatory HIV testing for children whose mothers were living with HIV. One of the most devastating fears of WLHA was that their children were being bullied by peers or that they faced expulsion from school.

Neighbors told people not to play with my children because their mother is HIV positive. When I heard that, I was very angry. I felt very sad…There was a time when my child went to kindergarten, the teacher did not allow it. The teacher kept saying that other parents did not agree for my child to study there…I brought a paper with negative test results, but the teacher wouldn’t listen. (Woman aged 38, middle school education, stable employment, one teenage child)

## Discussion

This study provides insight into the overarching challenges, including HIV-related stigma, lack of access to health information, and disparities in economic power and cultural expectations placed on women in Vietnam, and how these challenges influenced the interconnecting stages of WLHA’s reproductive journey, from family planning, pregnancy, perinatal care, and motherhood experiences. Based on these findings, the study underscores the importance of devising multifaceted strategies to improve WLHA’s support, services, and outcomes.

At an individual level, WLHA’s lack of knowledge about reproductive options, PMTCT precautions, and HIV prognoses resulted in notable distress. Given the positive association between health knowledge and self-efficacy [17], educating WLHA about current HIV treatment and PMTCT strategies and dispelling myths of poor HIV prognosis and the likelihood of having babies infected with HIV could empower WLHA to make well-informed reproductive decisions. Establishment of programs that offer feeding guidance for new WLHA mothers could also assuage fears of vertical transmission through breastmilk. Additionally, WLHA who could not bear children in this study experienced internalized stigma and shame. Cognitive behavioral stress management and expressive supportive therapy have been shown to enhance self-esteem while mitigating depression and anxiety specifically for WLHA [18]. Adapting a similar therapy strategy to Vietnam with the formation of peer groups for exchange of experiences could vastly improve mental health outcomes for WLHA.

On an interpersonal level, findings revealed how family plays a pivotal role in shaping the maternal health experiences of WLHA. Considering expectations for women to bear children, many WLHA in this study shared how they had to prioritize their husband’s or family’s desires before their own health and personal desires. The role of family in the reproductive decisions and experiences of WLHA was complex and varied. On one hand, some WLHA were fortunate to receive familial support with pregnancy and parenting. On the other hand, some WLHA grappled with stigma, alienation, and raising their children alone due to partner illness/separation and the traditional family-caring role in women. This experience is not unique to Vietnam as similar findings have also been reported in developed countries [6, 19]. Thus, family-centered care models where the family, especially the husband, are involved in decision-making, care planning, and child-rearing, can foster a sense of inclusion and responsibility from the whole family unit, and alleviate burden on WLHA [20, 21].

On a systems level, HIV-related stigma in healthcare settings significantly impacted WLHA’s entire reproductive experiences. Like previous studies, participants reported receiving discriminatory health counseling from their providers, such as advice for contraception and abortion rather than for feasible family planning options [22, 23]. During pregnancy, stigma towards pregnant WLHA resulted in delayed checkups and suboptimal prenatal care [24]. Consequentially, breakdown of patient-provider communication and failed interprofessional collaboration to implement PMTCT, resulted in HIV transmission to babies of WLHA [25]. Thus, specialized training to educate healthcare providers on management of pregnancies in WLHA is warranted. Ikeda and colleagues described a quality improvement approach across Southeast Asia, featuring routine measurement of stigma, team-based learning, and root cause analysis, to address multi-level drivers of stigma in healthcare settings [26]. Integration of HIV care with existing maternal and child health services has also been demonstrated to improve access, adherence, and retention to PMTCT services [27, 28]. These approaches could be adapted to tackle stigma in healthcare settings to ensure safety throughout WLHA’s perinatal experiences.

Finally, the reproductive and maternal health challenges faced by WLHA represent a salient socioeconomic and cultural issue. In Vietnam, like in many other countries, women typically have more limited access to resources, education, and labor market opportunities compared to men [29]. This gender disparity is exacerbated by HIV stigma and patriarchal norms, thereby intensifying the challenges WLHA face in their effort to gain economic stability and fulfill their societal and familial roles [30, 31]. This study also highlighted how societal stigma extends to children born to WLHA. These children faced isolation, bullying, and limited educational opportunities that could affect their futures. Potential strategies to address discrimination towards WLHA and their children include education campaigns to challenge stereotypes surrounding HIV, as well as enforcing policies and training on inclusivity and confidentiality practices at educational, employer, and healthcare institutions.

This study faced a few limitations. All participants were recruited from Hanoi, Vietnam’s capital city where attitudes are more liberal. Thus, these findings are not generalizable to WLHA in other areas with less open attitudes and resources. The interviews were also only conducted with WLHA, without the perspectives of their partners, families, or male counterparts. The data was self-reported and could be subject to social desirability bias. Finally, while pregnancies and deliveries are typically memorable events, it is important to note that some of our study participants experienced these during the 2000s and 2010s. Consequently, their recollections of perinatal experiences may be prone to recall bias. Additionally, these experiences might not completely reflect the present-day context, considering the advancements in PMTCT services, changes in health policies, and the growing social progressiveness in awareness of HIV/AIDS. Nonetheless, the findings from these interviews remain relevant today, as major challenges encountered by WLHA, including HIV-related stigma, gender disparities in social capital, traditional family roles, and inadequacies in patient confidentiality protection, continue to be evident in Vietnamese society [9, 31]. Moreover, the insights derived from these women’s experiences, even those from the past, contribute to a comprehensive narrative to enhance our understanding of the evolving landscape of perinatal care and motherhood among WLHA in Vietnam.

## Conclusions

In conclusion, this study highlights the intersection of HIV stigma, cultural beliefs, and gender norms that shape WLHA’s holistic reproductive experiences in Vietnam. Our findings underscore the need for multifaceted strategies at the individual, community, and societal levels to improve self-efficacy, social support, and medical care for WLHA throughout family planning, pregnancy, and motherhood. Policy and educational programs targeted for the WLHA, their families, healthcare providers, and the public are needed to promote access and equity for WLHA in Vietnam.

## Data Availability

Not applicable.

## Abbreviations

HIV: Human immunodeficiency virus
WLHA: Women living with HIV/AIDS
PMTCT: Prevention of mother to child transmission

## References

[1] WHO. Summary of the global HIV epidemic, 2021. World Health Organization; 2022 Jul. Available from: https://www.who.int/teams/global-hiv-hepatitis-and-stis-programmes/hiv/strategic-information/hiv-data-and-statistics.

[2] Griesbeck M, Scully E, Altfeld M (2016). Sex and gender differences in HIV-1 infection. Clin Sci.

[3] Moran JA, Turner SR, Marsden MD (2022). Contribution of sex differences to HIV immunology, pathogenesis, and cure approaches. Front Immunol.

[4] Edmonds A, Breskin A, Cole SR, Westreich D, Ramirez C, Cocohoba J (2021). Poverty, deprivation, and mortality risk among women with HIV in the United States. Epidemiology.

[5] Sohler NL, Li X, Cunningham CO (2009). Gender disparities in HIV health care utilization among the severely disadvantaged: can we determine the reasons?. AIDS Patient Care STDS.

[6] Huertas-Zurriaga A, Palmieri PA, Edwards JE, Cesario SK, Alonso-Fernandez S, Pardell-Dominguez L (2021). Motherhood and decision-making among women living with HIV in developed countries: a systematic review with qualitative research synthesis. Reprod Health.

[7] Yuvaraj A, Kundu P (2020). A snapshot of reproductive and sexual health and rights of women living with HIV in Asia. Oral Dis.

[8] UNAIDS. Country Fact Sheets Vietnam 2021 HIV and AIDS Estimates. UNAIDS; 2021. Available from: https://www.unaids.org/en/regionscountries/countries/vietnam

[9] Hong KT, Anh NTV, Ogden J (2004). Understanding HIV and AIDS-related stigma and discrimination in Vietnam.

[10] Oosterhoff P, Anh NT, Hanh NT, Yen PN, Wright P, Hardon A (2008). Holding the line: family responses to pregnancy and the desire for a child in the context of HIV in Vietnam. Cult Health Sex.

[11] Tran BX, Duong HD, Nguyen AQ, Pham LD, Tran TT, Latkin CA (2018). Child desire among men and women living with HIV/AIDS in the traditional culture of Vietnam. AIDS Behav.

[12] Hanh NT, Gammeltoft TM, Rasch V (2011). Number and timing of antenatal HIV testing: evidence from a community-based study in Northern Vietnam. BMC Public Health.

[13] Oosterhoff P, Hardon AP, Nguyen TA, Pham NY, Wright P (2008). Dealing with a positive result: routine HIV testing of pregnant women in Vietnam. AIDS Care.

[14] Messersmith LJ, Semrau K, Anh TL, Trang NNN, Hoa DM, Eifler K (2012). Women living with HIV in Vietnam: desire for children, use of sexual and reproductive health services, and advice from providers. Reprod Health Matters.

[15] Le CT, Vu TT, Luu MC, Do TN, Dinh TH, Kamb ML (2008). Preventing mother-to-child transmission of HIV in Vietnam: an assessment of progress and future directions. J Trop Pediatr.

[16] Ryan G, Bernard R (2003). Techniques to identify themes. Field Methods.

[17] Villegas N, Cianelli R, Gonzalez-Guarda R, Kaelber L, Ferrer L, Peragallo N (2013). Predictors of self-efficacy for HIV prevention among Hispanic women in South Florida. J Assoc Nurses AIDS Care.

[18] Jones DL, Owens MI, Lydston D, Tobin JN, Brondolo E, Weiss SM (2010). Self efficacy and distress in women with AIDS: the SMART/EST women’s project. AIDS Care.

[19] Moseholm E, Aho I, Mellgren Å, Johansen IS, Storgaard M, Pedersen G (2022). The experience of pregnancy among women living with HIV in Nordic countries: a qualitative narrative enquiry. Womens Health (Lond).

[20] Rochat TJ, Bland R, Coovadia H, Stein A, Newell M-L (2011). Towards a family-centered approach to HIV treatment and care for HIV-exposed children, their mothers and their families in poorly resourced settings. Future Virol.

[21] Triulzi I, Palla I, Ciccacci F, Orlando S, Palombi L, Turchetti G (2019). The effectiveness of interventions to involve men living with HIV positive pregnant women in low-income countries: a systematic review of the literature. BMC Health Serv Res.

[22] Chi BK, Rasch V, Hȧnh NTT, Gammeltoft T (2010). Induced abortion among HIV-positive women in Northern Vietnam: exploring reproductive dilemmas. Cult Health Sex.

[23] Tanner AE, Chambers BD, Philbin MM, Ware S, Eluka N, Ma A (2018). The intersection between women’s reproductive desires and HIV care providers’ reproductive health practices: a mixed methods analysis. Matern Child Health J.

[24] Turan JM, Nyblade L (2013). HIV-related stigma as a barrier to achievement of global PMTCT and maternal health goals: a review of the evidence. AIDS Behav.

[25] Lin C, Li L, Ji G (2018). Prevention of mother-to-child transmission of HIV services in China: a conversation between healthcare professionals and migrant women with HIV. Int J Healthc Manag.

[26] Ikeda DJ, Nyblade L, Srithanaviboonchai K, Agins BD (2019). A quality improvement approach to the reduction of HIV-related stigma and discrimination in healthcare settings. BMJ Glob Health.

[27] Brittain K, Teasdale CA, Ngeno B, Odondi J, Ochanda B, Brown K (2021). Improving retention in antenatal and postnatal care: a systematic review of evidence to inform strategies for adolescents and young women living with HIV. J Int AIDS Soc.

[28] Vrazo AC, Firth J, Amzel A, Sedillo R, Ryan J, Phelps BR (2018). Interventions to significantly improve service uptake and retention of HIV-positive pregnant women and HIV-exposed infants along the prevention of mother-to-child transmission continuum of care: systematic review. Trop Med Int Health.

[29] ILO. World Employment and Social Outlook Trends 2023. International Labour Organization; 2023. Available from: https://www.ilo.org/global/research/global-reports/weso/WCMS_865387/lang--en/index.htm.

[30] Rodrigo C, Rajapakse S (2010). HIV, poverty and women. Int Health.

[31] Tran BX, Than PQT, Tran TT, Nguyen CT, Latkin CA (2019). Changing sources of stigma against patients with HIV/AIDS in the rapid expansion of antiretroviral treatment services in Vietnam. Biomed Res Int.

